# Body Mass Index (BMI): A Screening Tool Analysis

**DOI:** 10.7759/cureus.22119

**Published:** 2022-02-11

**Authors:** Deepesh Khanna, Cadynce Peltzer, Payal Kahar, Mayur S Parmar

**Affiliations:** 1 Foundational Sciences, Dr. Kiran C. Patel College of Osteopathic Medicine, Nova Southeastern University, Clearwater, USA; 2 Department of Health Sciences, Florida Gulf Coast University, Fort Myers, USA

**Keywords:** health predictor, childhood obesity, muscle mass, waist hip ratio, mortality, hypercholesterolemia, waist circumference, diabetes, hypertension, body mass index: bmi

## Abstract

Body mass index (BMI), a measurement based on a person’s height and weight, allows the classification of individuals into categories such as obese or overweight. With these classifications, we can assess risk for hypertension, diabetes, cancer, hypercholesterolemia, and other chronic diseases. Furthermore, childhood BMI serves as a prediction method for health and disease later in life. Along with BMI, researchers also study waist circumference and waist-to-hip ratio in correlation with the above-mentioned chronic illnesses. This brief review explores the associations between body mass index, waist circumference, and the waist-hip ratio as measurements and their capability as predictors for persistent conditions like diabetes and hypertension.

## Introduction and background

Obesity is a major public health crisis in the United States [1]. More than two-thirds of the U.S. population is either overweight or obese [2,3]. Body mass index (BMI) is one of the ways to measure obesity in the population. Other ways to measure obesity include the waist-to-hip ratio, the percentage of the body or visceral fat, and waist circumference [4]. Body mass index (BMI) can be calculated via mathematical operations where height and weight values are used to estimate the health status of a person [5]. BMI as a measurement is typically used to gauge the risk of developing chronic conditions such as diabetes, hypertension, depression, and cancer [4-8]. The BMI calculation will fall within a numerical range, which places an individual into one of four categories. This data is used by researchers and physicians to educate patients and the public of potential health risks detected within a specified category [9]. Researchers continue to evaluate the correlation between BMI and long-standing illness as well as associations between waist circumference as a predictor of health [10]. Expansion to this research dives into the association of childhood BMI as childhood obesity is also on the rise [11]. This review is markedly concise yet highlights the importance of BMI as a screening tool for chronic disease and mortality and briefly introduces the idea of childhood BMI as a tool for predicting disease later in life. While BMI is seemingly a good indicator for studying correlation with chronic disease, the measurement tool does not come without limitations, as discussed in this review.

## Review

Methods of literature review

Scientific journal articles published in relation to BMI and distributed from 1994 to 2019 were uncovered through a PubMed database search and from various article references. The search term ‘body mass index’ was utilized, and the reported inquiry uncovered 30 articles that depict the connection between BMI and other metabolic conditions like diabetes, hypertension, and disease-related mortality. Furthermore, the search resulted in articles regarding the relationship between BMI, waist circumference, waist-hip ratio, youth BMI as a wellbeing indicator, and limitations of BMI as a health evaluation tool. Considering the evidence presented by previous research, the affiliation between BMI and chronic disease variables can be extrapolated to the general population.

Results and discussion

BMI and Diabetes

A U.S. study conducted by Chan et al. [12] evaluated 51,529 males ranging from 40-75 years old who work in healthcare. The research revealed that those with a BMI greater than 35 kg/m2 had an increased risk of developing type II diabetes compared to those with BMI’s lower than 23 kg/m2. While this research upholds the claim that obesity increases the risk for disease, it is significant to consider the relevance of the data considering it was collected more than two decades ago (1994). More recently, researchers have studied whether these outcomes are universal and comparable on a global and racial scale.

Another study, based in Turkey, evaluated diabetes risk factors in 26,499 individuals, where regional analysis was completed and spanned North, South, East, West, and Central regions [13]. Additionally, the analysis was grouped with data considering family history, family size, age, education, waist circumference, BMI, hypertension, smoking, and the number of meals eaten per day. Across all demographics studied, an increase in waist circumference by one standard deviation also increased the likelihood of a new diabetes diagnosis by 1.16-fold. By the same measures, the male cohort resulted in a 1.28-fold increase in the likelihood that the individual will also have a new diagnosis for diabetes.

Similarly, Satman et al. determined that BMI is a notable measure of diabetes risk [13]. This research states that an increase in BMI by one standard deviation (5.9kg/m2) results in an increased probability for type II diabetes by 1.16 for men and 1.09 for women.

A further analysis was completed in a multi-ethnic, longitudinal study based out of Canada, which involved 59,824 non-diabetic adults. This data concluded that the chance of developing diabetes increases at lower BMI values in South Asian individuals (24kg/m2), Black individuals (26kg/m2), and Chinese individuals (25kg/m2) compared to White individuals (30kg/m2). Thus, it is reasonable to conclude the above-mentioned racial groups have an increased likelihood of developing diabetes at a lower BMI than their White counterparts [14]. Understanding the relationship between race and BMI is a useful tool for physicians and patients to create a personalized care plan and discuss diabetic risk. More so, the findings can be extrapolated to a larger scale to address public health concerns.

While BMI is seemingly a good indicator of chronic diseases, such as type II diabetes, some researchers suggest that other measurement tools are more useful for this prediction. For instance, BMI was the least effective predictor for cardiovascular risk factors such as diabetes, hypertension, and dyslipidemia compared to other measurements like waist circumference, waist-to-hip, and waist-to-height ratios, in the study performed by Lee et al. [15]. This meta-analysis included 80,000 participants across 10 articles and discovered that waist-to-height ratio was profoundly better at predicting risk for the diseases aforementioned. This success can be attributed to the ability of the waist-to-height ratio to consider central adiposity, whereas BMI lacks this capability.

BMI and hypertension

Similar studies have been completed which analyze the relationship between body mass index and the probability of developing hypertension. Hu et al. [16] considered survey data from 17,441 Finnish individuals from 1982 to 1992, which evaluated participant height, weight, and heart rate, among other factors. The measures of BMI and risk of developing hypertension were analyzed using hazard ratios and follow-up exams to determine if predictions were correctly forecasted. Results revealed an increasing trend based on BMI (BMI <25= 1.00, 25-29.9= 1.18, and >30= 1.66). Thus, a larger BMI was linked with a greater occurrence of hypertension in this population. Furthermore, this study discovered that physical activity mitigated the risk of developing hypertension remarkably, even if the body mass index was higher than normal.

Gelber et al. [17] investigated 13,563 non-hypertensive subjects and again after 14 years to determine if there was a link between BMI at the first interaction and prevalence of hypertension 14 years later. Results illustrated that even participants whose BMI’s were considered high in the “normal” category proved an increased risk of developing hypertension compared to individuals in the lowest quintile (<22.5 kg/m2). In tandem with this finding was a positive trend in hypertension development when compared to BMI 14 years prior. The study noted that individuals with a BMI greater than 26.4kg/m2 had a 1.85 times greater risk of becoming hypertensive. Both studies evaluate the link between BMI and the risk of developing hypertension, a tool that can be further used to understand the etiologies of cardiovascular disease.

BMI and hypercholesterolemia

Obesity is often associated with a dyslipidemic state where the increased levels of serum triglycerides and low-density lipoprotein (LDL) cholesterol correlate with increased body weight. Gostynski et al. [18] completed an analysis of over 48,000 subjects aged 25-64 years old and studied the relationship between BMI and prevalence of hypercholesterolemia (≥6.5 mmol/l). The researchers discovered a statistically significant positive correlation between BMI and hypercholesterolemia for people in the 25-50 years age group, with the strongest effect on those in the 25-39 years age range.

Additionally, the Bogalusa Heart Study and Tershakovec et al. [19] determined a relationship between the development of childhood obesity in girls and persistent hypercholesterolemia. Between 1973 and 1991, 3179 children aged 5-14 years old completed a lipoprotein assessment and were evaluated against BMI and cholesterol serum normal values. It was found that girls with high cholesterol were more likely to have a greater increase in BMI than non-hypercholesterolemic girls as they developed. While this data seems to suggest that high cholesterol may be used as a predictor for obesity, it is still important to note the existence of the correlation at all.

This brings up a significant value in directing public health measures at young people, especially in school-age years, to encourage healthy development and decrease the risk of developing metabolic and cardiovascular disease.

BMI and mortality

A study performed in the U.K. by Bhaskaran et al. [20] assessed a national mortality database of 3,632,674 people to evaluate possible correlations between all-cause mortality and cause-specific mortality to body mass index (BMI). In this analysis, the researchers utilized a hazard ratio and performed sensitivity analyses to adjust for differences in age, sex, smoking, diabetes, and other variables. From the 3,632,674 individuals, 1,969,648 people were categorized as never-smokers, and this cohort will be used for the remainder of the data analysis to eliminate smoking status as a variable. Data revealed the association between BMI and all-cause mortality, communicable and non-communicable diseases, both in smokers and never-smokers, as a J-shaped curve. There is a higher associated risk of mortality outcomes with participants with a BMI above 25 kg/m2. It is significant to note the lowest risk for mortality falls at the 25kg/m2 measurement. Obesity was associated with a 4.2-year reduction in remaining life for a 40-year-old male never-smoker and a 3.5-year reduction in remaining life for a 40-year-old never-smoker female when compared to healthy weight individuals [20].

BMI and waist circumference

Both BMI and waist circumference are measurements used to assess patients for obesity status. In Chinedu et al. [21], these factors were critically compared to determine if they were correlated. This study looked at 489 Nigerian participants between the ages of 19 and 75 years for height, weight, and waist circumference. The findings demonstrated a positive relationship between waist circumference and BMI that proved to be statistically significant (r=0.75). In another study, Gierach et al. [22] found analogous results when analyzing 839 individuals between the ages of 32 and 80 who have a diagnosis of metabolic syndrome. Their data also showed a positive correlation between waist circumference and BMI (r=0.78).

Additionally, a BMI comparison study was performed by Romero-Corral et al. [23], where the body mass index was compared with the calculated value of body fat percentage from bioelectric impedance analysis (BIA). 13,601 Americans participated in a cross-sectional study which was meant to determine the efficiency of BMI to diagnose obesity when compared to the World Health Organization reference standard for obesity of BF% >25% in men and >35% in women. BMI and body fat percentage were studied by sex and age groups and adjusted for race. Ultimately, the results suggested that BMI has a high specificity yet a low sensitivity for detecting obesity, which means cases of obesity are often missed. Specifically, the researchers discovered that BMI is more tightly associated with high lean-body mass than body fat percentage (BF%) in men, and BMI is less reliable for diagnosing obesity in elderly populations. In opposition to these findings, Flegal et al. [24] found that for male participants, waist circumference was a better predictor over BMI at determining body fat percentage. Ultimately, it is still up for debate on which tool is profoundly more reliable as a measure of obesity, but both BMI and waist circumference have their proficiencies and limitations.

BMI and waist-to-hip ratio

Although waist circumference is a seemingly prognostic tool for obesity, another measurement value should be articulated- the waist-to-hip measurement ratio. When comparing four measures of obesity: waist circumference, BMI, waist to height ratio, and waist to hip ratio, Bener et al. [25] concluded that the best measure of obesity was waist circumference in both men and women. It was determined amongst male participants that there was a larger area under the curve (AUC) for waist-to-hip ratio than BMI, thus making waist-to-hip ratio the second-best indicator behind waist circumference for this population.

Dalton et al. [26] studied Australian participants and adjusted for age variables between BMI, waist circumference, and waist to hip ratio. Researchers determined there were no fundamental differences in any of the three measurement tools in predicting obesity and associated chronic disease. (Table 1)

**Table 1:** Studies evaluating a correlation between BMI and other Body Composition Evaluation Tools

Study, year	Population	Study Duration	Significant Findings
Chinedu et al. [21] 2013	489 Nigerian participants, ranging from 18-75 years old	April 2012 to May 2012	Positive and statistically significant correlation (r=0.75) between BMI and waist circumference (WC)
Gierach et al. [22] 2014	839 participants with metabolic syndrome, ranging from 32-80 years	24-month period, cross-sectional study	WC and BMI are correlated; significant- positive relationship (r=0.78)
Romero-Corral et al. [23] 2008	13,601 participants ranging from 20-79 years old	NHANES survey data integrated into cross-sectional study	BMI ≥ 30 kg/m2 has a higher specificity but lower sensitivity for VF% obesity.
Flegal et al. [24] 2008	12,901 participants ranging from 18 years or older	Data collected between 1999-2004; NHANES database	WC and BMI were better correlated with one another than BF%
Bener et al. [25] 2013	1,552 participants ranging from 20 years or older	April 2011- December 2012 integrated cross-sectional study	Best predictor of metabolic syndrome is WC, 2^nd^ best in males is WHR
Dalton et al. [26] 2003	11,247 participants ranging from 25 years or older	A cross-sectional study with data gathered in 2000	No fundamental difference between BMI, WHR, WC for evaluating obesity and chronic disease.

BMI and muscle mass

While BMI is a seemingly good measure for predicting obesity and determining risk for chronic disease, it is not entirely inclusive. Individuals with large amounts of muscle mass, such as athletes, may be inaccurately placed into an overweight or obese category based on height and weight. According to Kyle et al. [27], BMI alone is not sufficient at providing details on the contribution of fat-free mass to body weight. The study looked at the relationship between BMI and fat-free mass index (FFMI) and determined that 71% of the subjects with normal BMI had a normal FFMI as well. Additionally, they found five subjects out of 3533 subjects who were considered obese on the BMI scale but fell in the normal range for FFMI.

Additionally, it is previously known that skeletal muscle mass decreases and fat mass increases as a person age. Some elderly people claim normal or even low BMI scores when in reality, they have an increased fat-to-muscle ratio and should be evaluated as obese for secondary illnesses [28]. More so, muscle mass and body mass composition are dependent on a person’s sex assigned at birth. Since BMI is only weight/ height squared, there is no consideration for variations in fat mass between sexes or the effects of other complexities such as pregnancy, breastfeeding, cancer, or osteoporosis [29]. In a European study, researchers compared BMI calculations against DXA and densitometry measurements to determine if BMI was a good measure of body fat percentage. They noted that BMI calculations inaccurately categorized 7% of female and 8% of male participants into the obese category, where guidelines for obesity were >35% body fat for females and >25% body fat for males, respectively [30].

Childhood BMI as a predictor of health

All the aforementioned research studies and analyses found correlations between adult participants and BMI, but it is also important to note childhood BMI as a reliable predictor for chronic disease later in life. Twig et al. [31] studied teenagers in Israel to determine a potential relationship between BMI and cardiovascular-associated death in adulthood. The researchers uncovered a positive association between childhood BMI and cardiovascular death within the 50^th^ to 74^th^ percentile of the BMI range and noted an even stronger relationship with BMI above the 95^th^ percentile.

A plethora of studies found similar correlations between high childhood BMI and disease later in life. One, in particular, noticed a correlation between increased BMI and an increased incidence of type II diabetes in adult years [32]. Additionally, researchers considered childhood BMI as a potential predictor for endometrial cancer and noted that a higher BMI in girls is positively associated with the risk of developing endometrial cancer as an adult [33]. The same researcher, Aarestrup et al. [34]; however, found no relationship between childhood BMI and the probability of prostate cancer development in men once the data was adjusted for height. This is an important differentiation and springboard for future studies to analyze the difference in BMI prediction based on assigned sex at birth.

Along with the manifestation of adult disease, there are other factors like mental health and sleep that can be studied with BMI. One study investigated the link between high childhood BMI and suicidal behavior based on the presumption that individuals with high BMI values showed a trend of increased depression, a known predictor of suicidal behavior [35]. Collectively, this data reveals the importance of maintaining a healthy childhood BMI and the U.S. childhood obesity epidemic being monumental in the health of the population entirely. Pileggi et al. [36] looked at sleep in a population of 542 middle-school-aged children in southern Italy. They found that children with shorter sleep patterns have higher BMI (+0.77 kg/m2), advising that sleep should be encouraged to keep BMI in a healthy range. With this limited data, there is no way to tell if sleep or high BMI is the initial culprit causing the other variable, but likely this correlation is bidirectional.

Analysis of childhood BMI without also looking at potential etiology would be incomplete, so Bai et al. [37] collected data from over 2.5 million children and adolescents in the Texas school system and discovered that socioeconomic status (based on the eligibility for free or reduced lunch) was a significant predictor for high BMI in these children. Many factors can be studied to support this research in health disparities, including access and cost of healthy foods and the geographic location of low socioeconomic status (SES) homes in relation to food deserts.

Limitations of BMI as a health assessment tool

While BMI is a widely used measure, it does not come without limitations. BMI is a direct calculation that involves the measurements of height and weight, so it cannot assess the body fat percentage of an individual. This is particularly troubling for athletes and people with higher-than-average muscle mass [38]. A person with a high BMI who has a higher level of fat-free mass could be mistakenly categorized as overweight or obese. In contrast, a person with a larger volume of fat mass could be identified as healthier than predicted. There also may be a disparity in BMI measurement in determining obesity in genders, ages, and races [38]. One solution to this discrepancy is using BMI in combination with waist circumference to increase validity and accurate reporting of health status [39].

## Conclusions

BMI does correlate with health outcomes and cause-specific mortality, and measurements can start being recorded as early as childhood. However, using BMI to categorize obesity for the sake of predicting chronic disease risk is suboptimal, and other measures like waist circumference and waist to hip ratio may be preferred. Should BMI still be used for screening, it ought to be altered to consider variability in sex, age, and muscle mass or used in congruence with other measures to determine personalized treatment and nutrition counseling. 

## References

[1] Mardock M, Lockard B, Oliver J (2011). Comparative effectiveness of two popular weight loss programs in women I: body composition and resting energy expenditure. J Int Soc Sports Nutr.

[2] Baetge C, Earnest CP, Lockard B (2017). Efficacy of a randomized trial examining commercial weight loss programs and exercise on metabolic syndrome in overweight and obese women. Appl Physiol Nutr Metab.

[3] Tiwari A, Balasundaram P (2022). Public Health Considerations Regarding Obesity. StatPearls. Treasure Island.

[4] Khanna D, Rehman A (2022). Pathophysiology of obesity. StatPearls. Treasure Island.

[5] Oniszczenko W, Stanisławiak E (2019). Association between sex and body mass index as mediated by temperament in a nonclinical adult sample. Eat Weight Disord.

[6] Khanna D, Baetge C, Simbo S (2017). Effects of diet and exercise-induced weight loss in sedentary obese women on inflammatory markers, resistin, and visfatin. J Nutr Obes.

[7] Khatib M, Badillo N, Kahar P, Khanna D (2021). The risk of chronic diseases in individuals responding to a measure for the initial screening of depression and reported feelings of being down, depressed, or hopeless. Cureus.

[8] Adriana C, Claire B, Peter Murano (2016). Efficacy of commercial weight loss programs on metabolic syndrome. FASEB J.

[9] Clark DO, Mungai SM (1997). Distribution and association of chronic disease and mobility difficulty across four body mass index categories of African-American women. Am J Epidemiol.

[10] Levers K, Simbo S, Lockard B (2013). Effects of exercise and diet-induced weight loss on markers of inflammation I: impact on body composition and markers of health and fitness. J Int Soc Sports Nutr.

[11] Khanna D, Mutter CM, Kahar P (2021). Perception of overall health, weight status, and gaining weight in relationship with self-reported BMI among high school students. Cureus.

[12] Chan JM, Rimm EB, Colditz GA, Stampfer MJ, Willett WC (1994). Obesity, fat distribution, and weight gain as risk factors for clinical diabetes in men. Diabetes Care.

[13] Satman I, Omer B, Tutuncu Y (2013). Twelve-year trends in the prevalence and risk factors of diabetes and prediabetes in Turkish adults. Eur J Epidemiol.

[14] Chiu M, Austin PC, Manuel DG, Shah BR, Tu JV (2011). Deriving ethnic-specific BMI cutoff points for assessing diabetes risk. Diabetes Care.

[15] Lee CM, Huxley RR, Wildman RP, Woodward M (2008). Indices of abdominal obesity are better discriminators of cardiovascular risk factors than BMI: a meta-analysis. J Clin Epidemiol.

[16] Hu G, Barengo NC, Tuomilehto J, Lakka TA, Nissinen A, Jousilahti P (2004). Relationship of physical activity and body mass index to the risk of hypertension: a prospective study in Finland. Hypertension.

[17] Gelber RP, Gaziano JM, Manson JE, Buring JE, Sesso HD (2007). A prospective study of body mass index and the risk of developing hypertension in men. Am J Hypertens.

[18] Gostynski M, Gutzwiller F, Kuulasmaa K, Döring A, Ferrario M, Grafnetter D, Pajak A (2004). Analysis of the relationship between total cholesterol, age, body mass index among males and females in the WHO MONICA Project. Int J Obes.

[19] Tershakovec AM, Jawad AF, Stouffer NO, Elkasabany A, Srinivasan SR, Berenson GS (2002). Persistent hypercholesterolemia is associated with the development of obesity among girls: the Bogalusa Heart Study. Am J Clin Nutr.

[20] Bhaskaran K, Dos-Santos-Silva I, Leon DA, Douglas IJ, Smeeth L (2018). Association of BMI with overall and cause-specific mortality: a population-based cohort study of 3·6 million adults in the UK. Lancet Diab Endoc.

[21] Chinedu SN, Ogunlana OO, Azuh DE (2013). Correlation between body mass index and waist circumference in nigerian adults: implication as indicators of health status. J Public Health Res.

[22] Gierach J, Ewertowska M, Arndt A, Junik R (2014). Correlation between body mass index and waist circumference in patients with metabolic syndrome. Int Sch Res Not.

[23] Romero-Corral A, Somers VK, Sierra-Johnson J (2008). Accuracy of body mass index in diagnosing obesity in the adult general population. Int J Obes (Lond).

[24] Flegal KM, Shepherd JA, Looker AC (2009). Comparisons of percentage body fat, body mass index, waist circumference, and waist-stature ratio in adults. Am J Clin Nutr.

[25] Bener A, Yousafzai MT, Darwish S, Al-Hamaq AO, Nasralla EA, Abdul-Ghani M (2013). Obesity index that better predict metabolic syndrome: body mass index, waist circumference, waist hip ratio, or waist height ratio. J Obes.

[26] Dalton M, Cameron AJ, Zimmet PZ, Shaw JE, Jolley D, Dunstan DW, Welborn TA (2003). Waist circumference, waist-hip ratio and body mass index and their correlation with cardiovascular disease risk factors in Australian adults. J Intern Med.

[27] Kyle UG, Schutz Y, Dupertuis YM (2003). Body composition interpretation: contributions of the fat-free mass index and the body fat mass index. Nutrition.

[28] Rothman KJ (2008). BMI-related errors in the measurement of obesity. Int J Obes (Lond).

[29] Mazzoccoli G (2016). Body composition: where and when. Eur J Radiol.

[30] Deurenberg P, Andreoli A, Borg P (2001). The validity of predicted body fat percentage from body mass index and from impedance in samples of five European populations. Eur J Clin Nutr.

[31] Twig G, Yaniv G, Levine H (2016). Body-mass index in 2.3 million adolescents and cardiovascular death in adulthood. N Engl J Med.

[32] Zimmermann E, Bjerregaard LG, Gamborg M, Vaag AA, Sørensen TI, Baker JL (2017). Childhood body mass index and development of type 2 diabetes throughout adult life-a large-scale Danish cohort study. Obesity (Silver Spring).

[33] Aarestrup J, Gamborg M, Ulrich LG, Sørensen TI, Baker JL (2016). Childhood body mass index and height and risk of histologic subtypes of endometrial cancer. Int J Obes (Lond).

[34] Aarestrup J, Gamborg M, Cook MB, Sørensen TI, Baker JL (2014). Childhood body mass index and the risk of prostate cancer in adult men. Br J Cancer.

[35] Perera S, Eisen R, Bawor M, Dennis B, de Souza R, Thabane L, Samaan Z (2015). Association between body mass index and suicidal behaviors: a systematic review protocol. Syst Rev.

[36] Pileggi C, Lotito F, Bianco A, Nobile CG, Pavia M (2013). Relationship between chronic short sleep duration and childhood body mass index: a school-based cross-sectional study. PLoS One.

[37] Bai Y, Welk GJ (2017). School and county correlates associated with youth body mass index. Med Sci Sports Exerc.

[38] Buss J (2014). Limitations of body mass index to assess body fat. Workplace Health Saf.

[39] Aeberli I, Gut-Knabenhans M, Kusche-Ammann RS, Molinari L, Zimmermann MB (2013). A composite score combining waist circumference and body mass index more accurately predicts body fat percentage in 6- to 13-year-old children. Eur J Nutr.

